# The short inventory of grazing (SIG): development and validation of a new brief measure of a common eating behaviour with a compulsive dimension

**DOI:** 10.1186/s40337-019-0234-6

**Published:** 2019-02-07

**Authors:** Andreea I. Heriseanu, Phillipa Hay, Stephen Touyz

**Affiliations:** 1 School of Medicine, Western Sydney University, Locked Bag 1797, Penrith, 2751 NSW Australia; 2 School of Psychology, University of Sydney, Level 3, Building M02F, 94 Mallett St, Camperdown, 2050 NSW Australia

**Keywords:** Grazing, Obesity, Eating disorders, Compulsive eating, Scale validation, Loss of control over eating, Quality of life

## Abstract

**Background:**

Grazing, the repetitious and unplanned eating of small amounts of food with or without a sense of loss of control (LOC), is an eating pattern of recent interest which is highly prevalent in eating disorders and obesity. The current study aimed to (1) assess psychometric properties of a short inventory of grazing (SIG), consisting of a “grazing in general” item and a “compulsive/LOC grazing” item and (2) examine associations between compulsive and non-compulsive grazing and body mass index (BMI), eating disorder psychopathology, distress and health-related quality of life.

**Methods:**

Participants recruited from a university and the community (*n* = 227; 75.3% female; age = 25.00 (9.88; 17.58–57.17) years; BMI = 23.24 (4.91, 14.20–46.06) kg/m^2^) completed an online test battery including the SIG. Parametric and non-parametric statistics were computed to assess internal consistency, test-retest reliability and construct validity, to test associations between the SIG and the other study variables, and to examine between-group differences.

**Results:**

The SIG demonstrated appropriate psychometric properties. Results indicated that both grazing in general and low-frequency LOC grazing are common; however, LOC grazing of moderate-severe frequency and/or associated with marked distress is unusual. Frequency of LOC grazing, but not grazing in general, was significantly associated with higher BMI, psychological distress, compensatory behaviours and lower mental health-related quality of life. The presence of compulsive grazing was also associated with eating disorder caseness and binge-type eating disorder diagnostic groups.

**Conclusions:**

Results support the positioning of “compulsive” LOC grazing on a continuum of problematic eating. The SIG is a parsimonious measure of this eating pattern of emergent interest.

**Electronic supplementary material:**

The online version of this article (10.1186/s40337-019-0234-6) contains supplementary material, which is available to authorized users.

## Plain English summary

Grazing is the repetitive and unplanned eating of small amounts of food that sometimes includes a feeling of loss of control, i.e. that you cannot stop or resist eating. Grazing is common in eating disorders and obesity. This study aimed to (1) test a new, two-question measure of grazing in general and grazing with a sense of loss of control and (2) look at connections with related concepts. Two hundred and twenty seven university and community participants (mainly female, average age 25 years, average body mass index in the “healthy” range) completed questionnaires online. The Short Inventory of Grazing (SIG) suitably measured how often grazing takes place. Grazing was common, but grazing with loss of control that happened at least four times a week and/or which caused strong distress (upset) was unusual. Only grazing with loss of control was related to higher body mass index, likelihood of having an eating disorder, compensating for overeating, more psychological distress, and worse mental health-related quality of life. Grazing with a sense of loss of control seems to be an unhelpful eating behaviour.

## Background

Both obesity and eating disorders (ED) are global contributors to disability, morbidity and mortality [1, 2]. Thus far, research into eating behaviours present in ED and obesity has predominantly targeted binge eating [3], although eating patterns in both ED and obesity are highly diverse, and the full range of eating behaviours to be addressed in treatment is not clear at present [4]. Grazing is one other eating pattern of interest that has been identified in ED and obesity, and which is relatively common in these groups [5, 6]. For obese samples, mean pooled prevalence of grazing was found to be approximately 30% in weight loss treatment settings, and approximately 23% in the community; in ED samples seeking treatment, prevalence was approximately 60% [7]. Less is known about the prevalence of grazing in normative samples. A prior study surveying grazing presence and frequency in a non-clinical sample of university students indicated that approximately 90% of participants endorsed some grazing, with 21% grazing on approximately half the number of days over the past month, 12% grazing most days, and 5% grazing every day [8].

One of the reasons for the considerable variance in prevalence estimates is the lack of consistency regarding the features of grazing in the overall body of literature. However, recent research has coalesced around a definition based on expert consensus [9], characterising grazing as the repetitive eating of small/modest amounts of food in an unplanned manner, with two subtypes: “compulsive” grazing, which incorporates a sense of loss of control (LOC; i.e. a sense that one cannot resist grazing), and “non-compulsive” grazing, where a sense of LOC is absent. A recent systematic review of grazing in obesity and ED [7] suggests that where LOC is present, associations exist with lower mental health-related quality of life (MHRQoL), higher psychological distress, more severe ED psychopathology (such as lower body image, more severe and frequent binge eating), and less successful weight reduction treatment outcomes. In adult non-clinical samples, grazing associated with a sense of LOC has been found to be positively correlated with BMI and psychological distress [10] and to have stronger associations with eating disorder psychopathology than non-compulsive grazing [11]; additionally, very recently a significant positive association has also been found between compulsive grazing and food addiction severity (based on the Yale Food Addiction Scale, which uses a substance dependence disorder symptom model modified for eating behaviours) [12]. Although this is changing, there is still a paucity of information on this pattern of eating in community-based samples, ED samples, as well as in persons with overweight/obesity not seeking weight loss treatment, or seeking non-surgical treatment.

Given that clinical and research interest in grazing is relatively recent, few measures for this pattern of eating exist. The Grazing Questionnaire (GQ) [3] (English) and the Rep(eat)-Q [10] (Portuguese and Norwegian) are the only validated measures that have been specifically developed to examine this pattern of eating. The GQ measures the severity of grazing based on proportion of time spent engaging in aspects of this behaviour, and contains a “grazing” factor, and a “LOC” factor. The GQ, however, does not provide frequency of grazing occasions over a particular time period, e.g. on a weekly basis. The Rep(eat)-Q does collect frequency data, but only over the past four weeks, which is a relatively short time frame for establishing if an enduring pattern exists (e.g. the DSM-5 criteria for bulimia nervosa (BN)/binge eating disorder (BED) require a three-month history of binge eating, an eating behaviour which is also characterised by a sense of LOC [13]). Additionally, the Rep(eat)-Q is a 12-item measure, which is less convenient to use in screening batteries or epidemiological studies, where brevity is an important consideration.

Neither the GQ or the Rep(eat)-Q include information on whether this form of eating is associated with distress specifically related to grazing itself (conceptually similar to distress due to binge eating, which is a diagnostic criterion for BED), or if it is associated with a decrease in health-related quality of life (HRQoL), similar to ED behaviours [14, 15]. These considerations are especially pertinent in determining whether grazing with or without a compulsive element represents a psychopathological eating behaviour requiring specific clinical attention, a matter which is still unclear.

Therefore, the aims of the current study are: (1) to validate a short inventory of grazing (SIG) which includes a compulsive dimension, and which follows the DSM-5 diagnostic style in terms of defining grazing behaviour and specifying frequency over the past 3 months; and (2) to compare compulsive and non-compulsive grazing in terms of BMI, ED psychopathology, grazing-related distress, general psychological distress and HRQoL.

## Methods

### Design

This was a prospective, descriptive study. Dependent variables were participants’ scores on the various study measures, including the SIG, ED symptomatology, HRQoL, and BMI.

### Item development

The SIG items were developed after canvassing the literature for defining features of grazing [7, 9], and following discussion with ED experts (co-authors ST and PH). Prior expert consensus found that (1) “repetitive” and (2) “unplanned eating” of (3) “small/modest amounts of food” were the primary defining features of grazing. Hence, the definition of grazing used in the first item of the SIG, assessing grazing in general (GRG), is: “repeatedly (more than twice in the same time period during the day) picking or nibbling small amounts of food outside of planned meals and snacks.” Given the aim of investigating a “compulsive” subtype of grazing including a sense of LOC, a second item was developed (GRLOC), following the definition of compulsive grazing presented in Conceição et al. [9] (and subsequently used in Conceição et al. [10]), i.e. occasions of grazing associated with a sense of not being able to resist eating, or being driven/compelled to eat.

Frequency is rated for both items on a seven-point scale (0 to 6) ranging from “none at all” to “eight or more times per week”. This reflects a DSM-5 style categorisation of severity based on frequency of BE in BED and BN, i.e. mild: 1–3 episodes per week; moderate: 4–7 episodes per week; and severe/extreme: ≥ 8 episodes per week. The SIG has been included in Additional file 1.

### Participants

A non-clinical sample consisting of 227 participants was selected for this study, consisting of university students (*n* = 153) and general community members (*n* = 74). University students chose to participate in the study via an online research participation system for University of Sydney psychology students, while community participants had access to the study information and a link to the survey on community websites such as Gumtree Australia. A self-selected subset of student participants (*n* = 51) completed the SIG a second time, after an interval of two weeks, for test-retest reliability. As reimbursement, university participants received course credit; community participants did not receive reimbursement. In order to maximize sample heterogeneity, no restrictions were placed on inclusion characteristics (e.g. BMI, sex, etc.), however participant data were only included if participants completed basic demographics and the SIG at a minimum, which represented approximately 50% of the survey. Sixty-tree recorded responses did not meet this criterion, and there were seven repeated responses; hence, out of 297 total responses, 70 (23.57%) were considered invalid and were not used.

All participants resided in Australia, and were predominantly of Caucasian (50.7%) or Asian (41.0%) ethnicity; most participants (92.5%) had completed at least 12 years of education, and most (63.4%) were single. The majority were physically healthy; in terms of psychological health, 46 (20.3%) reported current anxiety, 35 (15.4%) current depression, and 15 (6.6%) a current ED. For full demographic and health-related sample characteristics see Additional file 2.

As the community and university participants differed on demographic variables such as age, BMI and sex distribution, initial analyses were carried out to determine if differences existed between these subgroups in terms of the study outcomes. No significant differences were found between the groups in terms of grazing frequency/severity distribution, association with ED diagnostic group, or ED caseness, and when examining associations between the SIG and the other study measures, the two groups yielded a similar pattern of results. Therefore, the groups were merged to increase power, and subsequent analyses were conducted on the pooled sample data.

The study was approved by the University of Sydney Human Research Ethics Committee, and online written informed consent was obtained from all participants.

### Measures

The following measures were included in the study survey:

#### Demographic questions and brief health screen

This questionnaire included sociodemographic items such as age, sex, ethnicity, education level, postcode (for estimating income, using the ABS Estimates of Personal Income for Small Areas) [16], and health screening items such as mental and physical health conditions, and weight loss attempts.

#### BMI

This was calculated as kg/m^2^ based on self-reported weight and height. This was then categorised using the WHO adult BMI classification [17].

#### Grazing distress

A single item assessing severity of distress regarding grazing was used. The question asked, “Is the grazing you experience usually associated with distress?” was answered on a four-point scale: “N/A as I do not graze”, “Not at all”, “Yes - a little” or “Yes - a lot”. This item was based on the DSM-5 distress criterion for BED, i.e. presence of “marked distress”; this was operationalised as the endorsement of the “Yes – a lot” option.

#### Depression Anxiety Stress Scale (DASS-21) [18]

This 21-item self-report measure consists of three subscales each containing seven items assessing depression (e.g. “I couldn’t seem to experience any positive feeling at all”), anxiety (e.g. “ I felt I was close to panic”) and stress (e.g. “ I felt that I was using a lot of nervous energy”) severity experienced over the previous week. Responses are rated on a four-point scale, ranging from “Did not apply to me at all” to “Applied to me very much, or most of the time”. Higher scores indicate higher symptom severity. Cronbach’s alpha for the study sample was .922, .804 and .871 for the depression, anxiety and stress subscales, respectively, and .937 for the total score.

#### Grazing Questionnaire (GQ) [3]

This seven-item self-report instrument measures the severity of grazing behaviour in general rather than over a particular time frame. Items (e.g. “Do you eat more or less continuously throughout the day or during extended parts of the day (e.g., all afternoon)?”) are answered on a five-point scale ranging from “Never” to “All of the time”. Higher scores indicate more severe grazing. The GQ encompasses two factors: a “grazing” factor and a “LOC over grazing” factor. Cronbach’s alpha for the study sample was .908 for the GQ, .897 for the grazing factor and .900 for the LOC factor.

#### Eating Disorder Examination-Questionnaire (EDE-Q) [19]

This self-report measure assesses ED psychopathology over the previous 28 days and produces frequency information on episodes of objective overeating (OOE), objective and subjective binge eating (OBE and SBE), compensatory behaviours and driven exercise, as well as a global eating psychopathology score, and four subscale scores (restraint, eating concern, shape concern, weight concern). Examples of items include: “Have you gone for long periods of time (eight hours or more) without eating anything at all in order to influence your shape or weight?”, and “Have you had a definite fear that you might gain weight?” Cronbach’s alpha for the study sample was .957 for the global score, and .834, .849, .927, .862 for the Restraint, Eating Concern, Shape Concern, and Weight Concern subscales, respectively.

#### Binge Eating Scale (BES) [20]

The BES is a 16-item questionnaire assessing the presence and severity of binge eating symptomatology. Each item presents a range of three to four statements regarding an aspect of binge eating (e.g. “I can control my impulses towards food” to “I feel totally unable to control my relationship with food and I try desperately to fight my impulses toward food.”) In the current study, Cronbach’s alpha for the BES was .922.

#### Dutch Eating Behaviour Questionnaire (DEBQ) [21]

The DEBQ is a 33-item instrument assessing emotional, external and restrained eating behaviour. Items are rated on a five-point scale ranging from “Never” to “Very often”. Only the Emotional Eating subscale (13 items; e.g. “Do you have a desire to eat when you are feeling lonely?”) and External Eating subscale (10 items; e.g. “ Do you eat more than usual, when you see others eating?”) were used in the current study; these exhibited Cronbach’s alpha of .956 and .876, respectively.

#### Loss of Control Over Eating Scale-Brief (LOCES-B) [22]

The LOCES-B is a seven-item scale assessing degree of LOC while eating (e.g. “I felt helpless about controlling my eating.”) Frequency of different aspects of LOC while eating in the past 4 weeks is rated on a five-point scale ranging from “Never” to “Always”. Item scores were averaged to generate a mean total score. In the current study, Cronbach’s alpha for the LOCES-B was .951.

#### Medical Outcomes Study Short Form (SF-12) [23]

The SF-12 is a 12-item measure of HRQoL (e.g.: “During the past four weeks, how much did pain interfere with your normal work, including both work outside the home and housework?”). Two summary scores are generated: the Mental Component Summary (MCS) reflecting MHRQoL and Physical Component Summary (PCS) reflecting physical health-related quality of life (PHRQoL). These range between 0 and 100 with a mean of 50 and standard deviation of 10, and with increasing values equating to better HRQoL. In this study, Cronbach’s alpha for the SF-12 was .816.

#### Self-Report Habit Index (SRHI) [24]

THE SRHI is a 12-item instrument that measures self-reported perceptions of habit strength for an identified behaviour (in the current study, grazing; e.g. “Grazing is something…I do automatically.”) Answers are rated on a seven-point scale ranging from “Strongly disagree” to “Strongly agree”. Responses were summed to create an overall score. Cronbach’s alpha for the SRHI was .962. A different scoring algorithm, utilising only four of the items, yields a “behavioural automaticity index” (SRBAI), which was also calculated; its Cronbach’s alpha was .940.

#### Social Desirability Scale (SDS-17) [25]

The SDS is a 17-item instrument assessing an individual’s propensity for portraying themselves in a favourable manner, and was included in this battery in order to capture potential bias in self-reporting of grazing behaviour. Items (e.g. “I sometimes litter.”) are endorsed on a dichotomous “true”-“false” scale. Higher scores indicate higher social desirability. In the current study Item 4 was omitted, as instructed by the creators of the scale, due to its lack of validity in certain populations [25]. The Kuder-Richardson 20 internal consistency coefficient for the present study was .685.

#### ED research classification

Based on symptoms endorsed on the EDE-Q, algorithms were designed to classify participants into groups defined by clusters of symptoms that were similar to those found in anorexia nervosa (AN), BN, BED-Broad (i.e. criteria A, D, E and F of DSM-5 BED), other specified feeding or eating disorder-AN (OSFED-AN; i.e. atypical AN), OSFED-BN (atypical BN), OSFED-BED (atypical BED) and OSFED-PD (purging disorder), similar to Hay et al. [26] and Berg et al. [27], using DSM-5 criteria. For these algorithms, see Additional file 3.

#### ED caseness

An additional method for distinguishing ED cases from non-cases was employed, based on that derived by Mond et al. [28]: (1) EDE-Q Global Score ≥ 2.3 AND (2) the occurrence of any OBEs, OR exercising for weight or shape reasons at least once per week.

### Procedure

The SIG, grazing distress item, and the other questionnaires describe above, were included in an online survey delivered using the Qualtrics online survey software and completed by the entire sample. Test-retest data for the SIG only was collected from a subset of 51 university participants, following an interval of two weeks.

The psychometric properties of the SIG were assessed by applying the criteria of adequacy for measurement properties as defined by Terwee et al. [29] and modified by Burton et al. [30].

### Statistical plan

Data were cleaned prior to analysis and inspected for erroneous values and for normality. As many of the study instruments measure atypical behaviour or symptoms (e.g. OBE, depression severity), many of the obtained data were not normally distributed; therefore, robust or nonparametric tests were used where this was the case, as described below. Alpha was set at .05, unless otherwise noted.

Internal consistency for the SIG was tested with Spearman-Brown coefficient, as well as Cronbach’s alpha; it has been demonstrated Cronbach’s alpha is robust to departures from normality in sample sizes of at least 100 [31], and that the Spearman-Brown coefficient is less biased than alpha for two-item measures [32]. Test-retest reliability was calculated using Wilcoxon’s Signed-Rank Test and Kendall’s tau-b.

Correlations between the SIG and related measures was calculated using Kendall’s tau-b association (for non-normally distributed data); effect sizes were based on Cohen’s guidelines for *r*. As Kendall’s tau-b is not directly interpretable and yields smaller values than *r*, a conversion was employed [33, 34] which provided the following cut-offs for effect size: small: tau = .06 (equivalent to *r* = .1); medium: tau = .19 (equivalent to *r* = .3); large: tau = .33 (equivalent to *r* = .5). Between-group differences were tested with one-way ANOVA, as this has been shown to be robust to violations of the normality assumption [35, 36], or where group variances were heterogeneous, Welch’s Test was used instead. Omega-squared was used for effect size in between-group comparisons, using the formula provided by Carroll et al. [37], as it has been shown to be less biased than eta-squared, especially in the presence of non-normality or heteroscedasticity [38–40]; effect size cut-offs of .01 for small, .06 for medium and .14 for large were used. Given the substantial number of validation measures used, alpha was set at .01 for correlations and between-group analyses, to limit the type I error rate.

Predictive validity was tested with multiple linear regression, using heteroscedasticity-consistent standard error estimators where required (i.e. where the homoscedasticity assumption was not met), using the RLM macro [41, 42]. Association between the presence of compulsive grazing and BMI categories/ED diagnostic groups was conducted using Pearson chi-square, or Fisher’s Exact Test for small (< 5) cell counts. All reported analyses were conducted using the IBM SPSS Statistics 22 program.

## Results

### Frequency of grazing and distress

Frequency of grazing as measured by SIG and the level of distress due to grazing is listed in Table 1. Most participants endorsed “mild” or “moderate” grazing (66.5%), with 14.5% endorsing “severe” grazing. One hundred and ninety-five participants (85.9%) had no or “mild” LOC grazing, with only 14.1% experiencing “moderate” or “severe” LOC grazing; 9.7% of participants endorsed marked distress due to grazing. Scores obtained by the sample on the study measures are recorded in Additional file 4.

**Table 1:** Frequency of grazing and distress due to grazing

		SIG 1 GRG Frequency (%)	SIG 2 GRLOC Frequency (%)	Grazing distress	Frequency (%)
Sample groups					
Total sample	No grazing	17 (7.5)	90 (39.6)	None	95 (41.9)
	< 1/wk	26 (11.5)	42 (18.5)	A little	93 (41.0)
	Mild (1–3)	80 (35.2)	63 (27.8)	A lot	22 (9.7)
	Moderate (4–7)	71 (31.3)	26 (11.5)		
	Severe (8+)	33 (14.5)	6 (2.6)		
University sample	No grazing	10 (6.5)	67 (43.8)	None	66 (43.1)
	< 1/wk	19 (12.4)	31 (20.3)	A little	65 (42.5)
	Mild (1–3)	57 (37.3)	34 (22.2)	A lot	12 (7.8)
	Moderate (4–7)	47 (30.7)	18 (11.8)		
	Severe (8+)	20 (13.1)	3 (2.0)		
Community sample	No grazing	7 (9.5)	23 (31.1)	None	29 (39.2)
	< 1/wk	7 (9.5)	11 (14.9)	A little	28 (37.8)
	Mild (1–3)	23 (31.1)	29 (39.2)	A lot	10 (13.5)
	Moderate (4–7)	24 (32.4)	8 (10.8)		
	Severe (8+)	13 (17.6)	3 (4.1)		

### Psychometric properties of the SIG

#### Internal consistency

Spearman-Brown split-half reliability coefficient, as well as Cronbach’s alpha, were obtained. The SIG displayed acceptable internal consistency (Spearman-Brown coefficient = 0.73; Cronbach’s alpha = 0.72). The two items displayed a strong association with each other (Kendall’s tau-b = .468, *p* < .001).

#### Reliability

Test-retest reliability was calculated for a university sample subset (*n* = 51) after an interval of two weeks. The results showed good test-retest reliability for the GRLOC item, with paired sample tests finding no significant difference between Time 1 and Time 2 scores (Wilcoxon Signed-Rank *Z* = −.54, *p* = .588). Scores on the GRG item, however, did show a significant difference between Time 1 and Time 2 (Wilcoxon Signed-Rank *Z* = − 3.19, *p* = .001). Associations between Time 1 and Time 2 scores were significant at *p* < .001 for both items: GRG Kendall’s tau-b = .82, GRLOC Kendall’s tau-b = .68.

#### Construct validity

SIG scores were not significantly associated with age or sex. Only GRLOC was positively associated with BMI (Kendall’s tau-b = .184, *p* < .001).

Convergent validity was assessed by calculating associations between the two SIG items and measures of ED psychopathology, BMI, and other theoretically-related constructs (see Table 2). Significant, positive associations were identified between the SIG and all included measures of ED symptoms (with the exception of restraint and compensatory behaviours, which were only significantly associated with GRLOC and not GRG); these were within the medium-to-large range for GRLOC, and small-to-medium range for GRG, indicating good convergent validity of the SIG.

**Table 2:** Associations between SIG items and demographic characteristics, psychological, weight and eating variables

	SIG1 - GRG	SIG2 - GRLOC
*n = 227*		
Age	.016	.083
Sex	−.106	−.067
BMI	.079	.184^a^
Distress due to grazing	**.205** ^a^	**.433** ^a^
DASS-21 D	.059	**.189** ^a^
DASS-21 A	.072	.177^b^
DASS-21 S	.069	**.207** ^a^
DASS-21 T	.071	**.220** ^a^
*n = 219*		
EDE-Q OOE	.172^b^	**.362** ^a^
EDE-Q OBE	**.228** ^a^	**.519** ^a^
EDE-Q SBE	.170^c^	**.410** ^a^
EDE-Q Vomiting	.087	**.189** ^c^
EDE-Q Laxative use	−.025	**.192** ^b^
EDE-Q Driven exercise	.076	.184^b^
*n = 226*		
EDE-Q Restraint	.107	**.356** ^a^
EDE-Q Eating Concern	**.221** ^a^	**.472** ^a^
EDE-Q Shape Concern	.166^b^	**.371** ^a^
EDE-Q Weight Concern	.166^b^	**.392** ^a^
EDE-Q Global	.166^b^	**.422** ^a^
*n = 213*		
BES	**.299** ^a^	**.589** ^a^
*n = 210*		
DEBQ Emotional	**.279** ^a^	**.498** ^a^
DEBQ External	**.385** ^a^	**.405** ^a^
*n = 215*		
LOCES	**.327** ^a^	**.606** ^a^
SHRI Total	**.542** ^a^	**.431** ^a^
SHRI BAI	**.478** ^a^	**.427** ^a^
GQ Grazing	**.609** ^a^	**.466** ^a^
GQ LOC Grazing	**.374** ^a^	**.629** ^a^
GQ Total	**.556** ^a^	**.589** ^a^
SF-12 PCS	.018	−.001
SF-12 MCS	−.031	**-.215** ^a^
*n = 209*		
SDS17 (16-item)	−.092	-.177^b^

Note. ^a^ *p* < .001;^b^ *p* = .001; ^c^ *p* = .002. Associations represent Kendall tau-b values. Bold figures indicate significant associations with a medium or larger effect size

The SIG demonstrated good divergent validity; as expected, only GRLOC, and not GRG, was significantly and positively associated with psychological distress and negatively associated with MHRQoL and social desirability. Consistent with previous research on grazing, no associations were found between either of the SIG items and PHRQoL.

The SIG demonstrated good concurrent validity; associations with the GQ and its two factors [3] yielded significant positive associations in the large range. Associations were, however, < 0.80, which indicates that although the two questionnaires are highly related, they are not identical.

ANOVAs were performed to determine if the SIG could differentiate between subgroups of participants who were classified as ED cases and non-cases using the criteria determined in Mond et al. [28], participants who did and did not meet research criteria for an ED diagnosis based on EDE-Q responses, and participants whose BMIs fell into the “obese” range vs the “non-obese” range. SIG scores (both GRG and GRLOC) were significantly higher for participants classified as cases (GRG: 3.76 (±1.62) vs. 3.10 (±1.78), *F*(1, 221) = 6.45, *p* = .012, omega-sq. = 0.025; GRLOC: 2.71 (±1.64) vs. 1.06 (±1.37), Welch statistic (1, 95.20) = 49.59, *p* < .001, omega-sq. = 0.180). Similarly, SIG scores were significantly higher for participants who met research criteria for an ED diagnosis than those who did not (GRG: 3.90 (±1.70) vs. 2.87 (±1.69), *F*(1, 217) = 19.27, *p* < .001, omega-sq. = 0.077; GRLOC: 2.70 (±1.70) vs. 0.77 (±1.05), Welch statistic (1, 120.67) = 87.03, *p* < .001, omega-sq. = 0.282). GRG scores were not significantly different for participants with and without obesity (3.65 (±1.87) vs. 3.28 (±1.75), *F*(1, 225) = .83, *p* = .365), and GRLOC scores for these two groups were marginally significantly different (2.15 (±1.93) vs. 1.47 (±1.62), *F*(1, 225) = 3.09, *p* = .080, omega-sq. = 0.009).

#### Floor and ceiling effects

As seen in Table 1, < 15% of participants achieved the lowest or highest possible scores on either item of the SIG, suggesting that the scale used is able to reflect different frequency levels of this behaviour in the sample.

### Compulsive vs non-compulsive grazing

Since GRG as defined in Item 1 of the SIG encompasses both LOC grazing and non-LOC grazing, further analyses were undertaken to compare the participants who only engaged in non-LOC grazing (GRLOC-; *n* = 73) and those who engaged in any degree of LOC grazing (GRLOC+; *n* = 137). Significant differences were found on most variables as outlined below, and effect sizes for these differences were generally in the medium to very large range. Full results of one-way ANOVAs/Welch Tests comparing these two groups are presented in Additional file 5.

Age was not found to significantly differ between the groups (*p* = .115), and no significant association with sex was found (X^2^(1) = 0.012, *p* = .914).

#### Compulsive grazing and BMI

The GRLOC+ group had a higher BMI (closer to the “overweight” range) than the GRLOC- group (24.13 (±5.03) vs 21.82 (±3.65), *p* = .001). Further analyses were conducted to test if the presence of compulsive grazing was predictive of a BMI increase. Controlling for age, sex, household income, marital status and education, as well as for presence of OBEs/SBEs, the presence of GRLOC+ did significantly predict an increase in BMI of 1.14 kg/m^2^ over GRLOC- (95% CI [0.05, 2.23], SE = 0.55, *t*(194) = 2.06, *p* = .041). The severity of GRLOC also significantly predicted an increase in BMI, such that for every increase in severity category, BMI was predicted to increase by 1.29 kg/m^2^ (95% CI [0.09, 2.49], SE = 0.61, *t*(211) = 2.12, *p* = .035).

No significant association was found between compulsive grazing and the presence of a BMI above the “healthy” range (X^2^(1) = 2.28, *p* = .131), or the presence of obesity specifically (X^2^(1) = 1.37, *p* = .243). To assess this descriptively, BMI was examined across grazing severity levels; for all GRG severity levels, as well as for mild or moderate GRLOC, mean BMI was in the “healthy” range. For severe LOC grazing, mean BMI fell into the “obese” range (31.55 (±10.77) kg/m^2^), however, this group only contained six cases.

#### Compulsive grazing and ED

The GRLOC+ group experienced more severe eating psychopathology (including higher frequency and severity of BE, *p* < .001; more severe emotional and external eating, *p* < .001; and more severe LOC over eating, *p* < .001), and higher grazing habitualness (*p* < .001) than the GRLOC- group. No differences were found for compensatory behaviours (*p* > .01 for all).

In terms of research ED diagnoses, there was a statistically significant association between the presence of GRLOC+ and EDE-Q BN (X^2^(1) = 9.23, *p* = .002, phi = .210) and EDE-Q BED-Broad (X^2^(1) = 11.77, *p* = .001, phi = .241), and the association with EDE-Q OSFED-BED reached marginal significance (X^2^(1) = 3.79, *p* = .052, phi = .137). There was also a statistically significant association between the presence of grazing with LOC and EDE-Q ED caseness (X^2^(1) = 22.44, *p* < .001, phi = .330). There were no statistically significant association between the presence of grazing with LOC and EDE-Q AN (Fisher’s Exact Test *p* = .544), EDE-Q OSFED-AN (X^2^(1) = 1.14, *p* = .285) or EDE-Q OSFED-PD (Fisher’s Exact Test *p* = 1.000). Tests of association could not be performed for EDE-Q OSFED-BN, as none of the participants classified into this group endorsed any grazing.

#### Compulsive grazing, distress and HRQoL

The GRLOC+ group experienced higher distress related to grazing (*p* < .001), and specifically, a significant association existed with marked distress due to grazing (X^2^(1) = 9.89, *p* = .002, phi = .217). The compulsive grazing group also endorsed more severe psychological distress in general (largely in the “mild” range, vs. “normal” range for GRLOC-, *p* < .01).

The GRLOC+ group experienced lower MHRQoL than GRLOC- (38.09 (±12.25) vs 44.42 (±11.28), *p* < .001). Further analyses were conducted to test if compulsive grazing was predictive of a lower MHRQoL. Controlling for demographic variables (age, sex, marital status, education, household income and BMI), the presence of GRLOC+ significantly predicted a decrease in MHRQoL of 4.85 points over GRLOC- (95% CI [− 8.51, − 1.20], SE = 1.85, *t*(191) = − 2.62, *p* = .009). The severity of GRLOC also significantly predicted a decrease in MHRQoL, such that for every increase in severity category, MHRQoL was predicted to decrease by 4.12 points (95% CI [− 6.49, − 1.75], SE = 1.20, *t*(207) = − 3.43, *p <* .001).

## Discussion

The SIG showed adequate psychometric properties in a nonclinical sample consisting of university students and community members. Overall, the SIG was found to be a valid and reliable measure with adequate internal consistency across the whole sample. The SIG displayed good floor and ceiling effects. The GRLOC item displayed good test-retest reliability across an interval of two weeks, whereas the GRG item was found to show temporal variance across this interval. This suggests that LOC grazing is a more stable and enduring construct, whereas grazing without LOC varies over time, potentially in response to environmental variables.

The significant positive associations between the SIG and the GQ support the concurrent validity of the SIG. As expected, the GRG item was more highly associated with the “grazing” factor than the “LOC” factor of the GQ, whereas the GRLOC item displayed the opposite pattern. Good convergent and divergent validity were evidenced by the pattern of associations between SIG scores and the other relevant constructs. Both grazing in general and compulsive grazing scores were significantly higher for participants meeting criteria for ED caseness/research ED diagnosis, and both showed associations with related facets of ED psychopathology, although in all cases the associations were stronger for compulsive grazing than for grazing in general. Only compulsive grazing (GRLOC) was significantly and positively associated with BMI, psychological distress, restraint and compensatory behaviours, and negatively with MHRQoL and social desirability. Neither type of grazing was significantly associated with PHRQoL, which is consistent with prior research [7]. Interestingly, grazing in general and compulsive grazing showed similar associations with a measure of habitualness of grazing behaviour, suggesting the grazing with or without a compulsive element may possess an automatic, reflexive component, which is theoretically related to the repetitive nature of this form of eating. This result is consistent with prior research which suggests that grazing is related to experiencing less mindfulness [43], and specifically, eating which is less mindful [44].

When specifically examining differences between participants who engaged in compulsive grazing and participants who grazed without LOC, the compulsive grazing group had a significantly higher BMI than those without LOC, and compulsive grazing remained predictive of a higher BMI even when controlling for relevant demographic and eating variables. This finding is in line with other studies which distinguished between grazing with and without LOC (e.g. [10]). Previous studies which only assessed grazing generically (i.e. similar to the GRG item) have generally failed to find an association between grazing and BMI (e.g. [8, 45]). It must be noted, however, that the observed BMI difference between the two groups was not very large, and both groups had a mean BMI within the “healthy” range. Further, no significant association was found between the presence of compulsive grazing and the presence of overweight or obesity; however, there were only 20 participants with obesity (8.8%) in the sample, which is a significantly lower proportion that that found in the general population (27.9%), or even the inner-city Sydney rate (16.0%) [46], which may account for this finding. Participants with severe LOC grazing were the only subgroup with a mean BMI within the “obese” range, suggesting a link between high LOC grazing frequency and detrimental weight outcomes (although there were only six participants in this group, so this finding should be interpreted with caution).

The compulsive grazing group obtained elevated scores on most measures of ED psychopathology, whereas scores for the non-compulsive grazing group were largely consistent with general population norms [28, 47–49]. When examining associations with specific ED diagnostic groups, the presence of GRLOC was significantly associated with the predominantly binge-type ED diagnostic groups (BN, BED-Broad, and OSFED-BED; associations with OSFED-BN could not be computed). This is partly consistent with previous research in clinical ED groups seeking treatment [5] which found a higher prevalence of grazing in patients with BN than in those with BED and AN. Prior research used a definition of grazing which did not explicitly include or exclude LOC as mentioned above, which may account for the discrepancy with regards to the BED vs AN findings.

Few people (*n* = 17) did not engage in any grazing, a result consistent with other studies examining grazing in non-clinical samples [3, 8]. Hence, from a statistical infrequency perspective, grazing in general appears to be a normative behaviour. Interestingly, more than half the sample (60.4%) experienced some degree of LOC related to grazing, however most of these participants (76.6%) had LOC grazing of very low frequency. Only 32 participants (14.1% of the total sample, or 23.4% of those with any LOC grazing) experienced moderate-to-severe LOC grazing, and only 22 participants (9.7% of the sample) experienced marked distress regarding grazing behaviour. The significant, negative association between GRLOC and social desirability (similar to individuals who experience binge eating, or emotional and external eating [50, 51]) indicates that compulsive grazing may be under-reported, due to motivation to give socially desirable responses regarding “appropriate” eating behaviour.

Overall, the findings of this study support the notion of compulsive grazing, a type of grazing characterised by a sense of loss of control coupled with repetitive, unplanned eating of small amounts of food, as distinct from non-compulsive grazing. Previous research has not found clear support for classing compulsive grazing as a disordered eating behaviour, given relatively weak associations with psychological distress and other relevant constructs such as impulsivity [10]. The current study extended this research by examining grazing frequency over a longer period of time, and in conjunction with both general psychological distress and distress related to grazing, as well as quality of life and ED diagnostic grouping. Results reflected moderate negative correlations with psychological distress, MHRQoL scores similar to those found in a community sample meeting criteria for BED [26] and to normative scores for community members diagnosed with a mental health condition [52], and a significant association with marked distress specifically due to grazing, as well as with binge-type ED diagnostic groups. These findings suggest that compulsive grazing may be considered as part of a disordered eating pattern; whether it represents a stand-alone psychopathological eating behaviour, however, is not yet clear. Further investigation into the use of severity cut-offs is also required, as the current research obtained mixed results in this respect (only “severe” compulsive grazing appeared to be associated with a BMI in the “obese” range, whereas compulsive grazing of any frequency, i.e. even mild in severity, was associated with marked distress due to grazing, with lower MHRQoL, and with ED caseness/research diagnoses).

This study had several limitations, including a validation sample which was somewhat homogeneous in terms of demographics (predominantly female, young, physically healthy, with at least 12 years of education, with a mean BMI in the “healthy” range), although the inclusion of both students and general community members increased the variability of the sample. The SIG was not tested for face validity or for comprehension; however, a definition of grazing was provided as part of the SIG, to ensure that participants understood the characteristics of this eating pattern. ED diagnostic groups were based on self-report, which is not as valid as a clinical interview conducted by a mental health clinician (although studies have found that the EDE-Q can discriminate well between cases and non-cases [28]). Weight and height were also self-reported; while weight, more than height, is over- or under-reported in some cases, there is evidence that there is high concordance between self-reported and objectively measured weight [53]. Additionally, the MHRQoL of the overall sample was somewhat lower than the general population norms, and a relatively high proportion of the sample endorsed mental health conditions.

The study also demonstrated several strengths, such as the adequate sample size relative to the length of the SIG and the large range of validation measures included. The development of the SIG included expert consultation, and its wording and resulting severity categories are in line with DSM-5 disorders such as BN and BED. The SIG is a concise and easy-to-administer measure developed and validated in English which parsimoniously records frequency of both grazing in general, and grazing with a compulsive element.

Future research involving the SIG should involve larger samples, to establish population-level information about grazing with and without a compulsive dimension. Longitudinal research would also be of interest for examining changes in grazing behaviour over time, especially to determine if a pattern similar to that of binge eating emerges (i.e. increased prevalence over time, accompanied by a decrease in distress and functional impairment, as seen in Mitchison et al. [54]). The SIG should also be examined in clinical groups, such as individuals with obesity presenting for weight reduction treatment, and ED samples where a diagnosis of ED has been established by clinical interview. It would also be important to examine this eating behaviour in children and adolescents, as detrimental eating patterns develop at a young age and are associated with risk of developing an ED later in life [55]. Examining grazing within different ethnic groups would also be of interest, especially taking acculturation into account, as this has shown a differential impact on eating disorder psychopathology [56]. As a relatively new eating behaviour of interest, grazing should be assessed using various research methodologies (i.e. not only self-report), such as in laboratory studies and ecological momentary assessment studies. In addition, in order to better characterise grazing, associations with other processes should be examined; for example, a growing body of literature has suggested executive functioning deficits in individuals engaging in binge eating [57–59]. At present, it is unknown if similar deficits exist in persons who graze, especially where a LOC element is present. Additionally, since both grazing in general and compulsive grazing have shown an association with habitualness, and since theoretically the repetitive eating which characterises grazing could be seen as an automatic behaviour partly prompted by environmental cues, further exploration of the role of habit in maintaining this style of eating should be undertaken.

## Conclusions

In conclusion, the current study adds support to notion of compulsive grazing being part of a disordered eating continuum. The results of the study suggest that the SIG is a promising self-report measure of presence and frequency of grazing with or without a compulsive element, assessed over a longer period of time. Due to its parsimony, the SIG would be suitable for inclusion in epidemiological studies, where survey length and respondent burden are significant considerations. Inclusion of both SIG items would allow for differentiation and comparisons between compulsive and non-compulsive grazing. Given its pattern of associations with measures of ED psychopathology, distress and MHRQoL, and especially given the associations with binge-type EDs, the GRLOC item of the SIG could be considered for inclusion in ED screening, and for assessing a fuller range of eating behaviours which may be present in persons with ED or high BMI presenting for treatment.

## Additional files

Additional file 1: Instrument: Short Inventory of Grazing. (DOCX 15 kb)

Additional file 2: Sample demographics. (DOCX 20 kb)

Additional file 3: Additional method section: derivation of ED diagnostic groups based on the EDE-Q. (DOCX 17 kb)

Additional file 4: Additional results: scores obtained by the total sample on study measures of distress, HRQoL, eating psychopathology and related constructs. (DOCX 15 kb)

Additional file 5: Additional results: Differences in psychological, eating psychopathology and HRQoL scores between grazing participants with and without LOC grazing. (DOCX 18 kb)

## Abbreviations

AN: anorexia nervosa
BED: binge eating disorder
BES: Binge Eating Scale
BMI: body mass index
BN: bulimia nervosa
DASS-21: Depression Anxiety Stress Scale-21
DEBQ: Dutch Eating Behaviour Questionnaire
ED: eating disorder
EDE-Q: Eating Disorder Examination-Questionnaire
GQ: Grazing Questionnaire
GRG: grazing in general
GRLOC: grazing with a sense of loss of control
HRQoL: health-related quality of life
LOC: loss of control
LOCES: Loss of Control Over Eating Scale
MHRQoL: mental health-related quality of life
OBE: objective binge eating
OOE: objective overeating
OSFED: other specified feeding or eating disorder
PD: purging disorder
PHRQoL: physical health-related quality of life
SBE: subjective binge eating
SDS-17: Social Desirability Scale-17
SF-12: Short Form 12
SIG: Short Inventory of Grazing
SRBAI: Self-Report Behavioural Automaticity Index
SRHI: Self-Report Habit Index
