# Supplementary material for: The short inventory of grazing (SIG): development and validation of a new brief measure of a common eating behaviour with a compulsive dimension
Source: J Eat Disord. 2019 Feb 7;7:4. doi: 10.1186/s40337-019-0234-6 (PMC6366119; doi:10.1186/s40337-019-0234-6)
Supplement: Supplementary file 1 — Instrument: Short Inventory of Grazing. (DOCX 15 kb) [file 40337_2019_234_MOESM1_ESM.docx]

**Short Inventory of Grazing (SIG)**

Heriseanu AI, Hay P & Touyz S

1. I would like to ask you about occasions of grazing. By "grazing" I mean ***repeatedly*** (more than twice in the same time period during the day) picking or nibbling small amounts of food ***outside of planned meals and snacks.***

Over the ***past 3 months***, typically how many episodes of grazing have you had per week?

| None at all | Less than weekly | Once a week | Two to three times a week | Four to five times a week | Six to seven times a week | Eight or more times a week |
| --- | --- | --- | --- | --- | --- | --- |
| 0 | 1 | 2 | 3 | 4 | 5 | 6 |
|  |  |  |  |  |  |  |

1. This question refers to any occasions of grazing where you felt ***you have lost control over your eating*** (i.e. a feeling that you could not avoid going back to graze on food even if trying to resist, or felt compelled or driven to graze).

Over the ***past 3 months***, typically how many episodes of grazing with loss of control have you had per week?

| None at all | Less than weekly | Once a week | Two to three times a week | Four to five times a week | Six to seven times a week | Eight or more times a week |
| --- | --- | --- | --- | --- | --- | --- |
| 0 | 1 | 2 | 3 | 4 | 5 | 6 |
|  |  |  |  |  |  |  |

**Severity category**

| None or less than weekly | - |
| --- | --- |
| 1-3 times a week | Mild |
| 4-7 times a week | Moderate |
| 8 or more times a week | Severe |
