# Supplementary material for: The short inventory of grazing (SIG): development and validation of a new brief measure of a common eating behaviour with a compulsive dimension
Source: J Eat Disord. 2019 Feb 7;7:4. doi: 10.1186/s40337-019-0234-6 (PMC6366119; doi:10.1186/s40337-019-0234-6)
Supplement: Supplementary file 2 — Sample demographics. (DOCX 20 kb) [file 40337_2019_234_MOESM2_ESM.docx]

**Additional File 1 - Sample demographics**

|  |  | Total sample N = 227 | University  n = 153 | Community n = 74 | Test-retest sample n = 51 |
| --- | --- | --- | --- | --- | --- |
| Sex: n(%) | Female | 171 (75.3%) | 127 (83.0%) | 44 (59.5%) | 39 (76.5%) |
|  | Male | 56 (24.7%) | 26 (17.0%) | 30 (40.5%) | 12 (23.5%) |
|  | Other | 0 (0.0%) | 0 (0.0%) | 0 (0.0%) | 0 (0.0%) |
|  |  |  |  |  |  |
| Age: years  (M(SD; range)) |  | 25.00 (9.88; 17.58-57.17) | 20.00 (4.22; 17.58-46.50) | 35.33 (10.22; 18.25-57.17) | 19.45 (3.10; 17.67-39.00) |
|  |  |  |  |  |  |
| BMI: kg/m^2^  (M(SD; range)) |  | 23.24 (4.91; 14.20-46.06) | 21.92 (3.27; 15.50-35.16) | 26.27 (6.28; 14.20-46.06) | 22.46 (4.01; 15.50-35.16) |
|  |  |  |  |  |  |
| BMI category: n(%) | <18.5 | 22 (9.7%) | 18 (11.8%) | 4 (5.4%) | 5 (9.8%) |
|  | 18.5-24.99 | 153 (67.4%) | 118 (77.1%) | 35 (47.3%) | 37 (72.5%) |
|  | 25-29.99 | 32 (14.1%) | 11 (7.2%) | 21 (28.4%) | 5 (9.8%) |
|  | ≥30 | 20 (8.8%) | 6 (3.9%) | 14 (18.9%) | 4 (7.8%) |
|  |  |  |  |  |  |
| Ethnicity: n(%) | Asian | 93 (41.0%) | 83 (54.2%) | 10 (13.5%) | 26 (51.0%) |
|  | Caucasian | 115 (50.7%) | 53 (34.6%) | 62 (83.8%) | 19 (37.3%) |
|  | Other | 19 (8.4%) | 17 (11.1%) | 2 (2.8%) | 6 (11.8%) |
|  |  |  |  |  |  |
| Education: n(%) | University | 81 (35.7%) | 25 (16.4%) | 56 (75.7%) | 9 (17.7%) |
|  | Trade | 13 (5.7%) | 2 (1.3%) | 11 (14.9%) | 1 (2.0%) |
|  | Year 12/HSC | 129 (56.8%) | 123 (80.4%) | 6 (8.1%) | 40 (78.4%) |
|  | Other | 3 (1.3%) | 3 (2.0%) | 1 (1.4%) | 1 (2.0%) |
|  |  |  |  |  |  |
| Marital: n(%) | Relationship/ married | 77 (33.9%) | 35 (22.9%) | 42 (56.8%) | 9 (17.6%) |
|  | Single | 144 (63.4%) | 117 (76.5%) | 27 (36.5) | 42 (82.4%) |
|  | Other | 6 (2.6%) | 1 (0.7%) | 5 (6.8%) | 0 (0.0%) |
| Lifetime medical condition: n(%) | Yes | 29 (12.8%) | 16 (10.5%) | 13 (17.6%) | 2 (3.9%) |
|  | No | 198 (87.2%) | 137 (89.5%) | 61 (82.4%) | 49 (96.1%) |
| Psych. condition: n(%) |  |  |  |  |  |
| Anxiety | Current | 27 (11.9%) | 11 (7.2%) | 16 (21.6%) | 3 (2.9%) |
|  | Past | 30 (13.2%) | 22 (14.4%) | 8 (10.8%) | 10 (9.8%) |
|  | Current and past | 19 (8.4%) | 10 (6.5%) | 9 (12.2%) | 3 (2.9%) |
| Depression | Current | 17 (7.5%) | 4 (2.6%) | 13 (17.6%) | 1 (1.0%) |
|  | Past | 36 (15.9%) | 21 (13.7%) | 15 (20.3%) | 6 (5.9%) |
|  | Current and past | 18 (7.9%) | 8 (5.2%) | 10 (13.5%) | 4 (3.9%) |
| Other non-ED | Current | 11 (4.8%) | 2 (1.4%) | 9 (12.2%) | 0 (0.0%) |
|  | Past | 11 (4.8%) | 6 (3.9%) | 5 (6.9%) | 0 (0.0%) |
|  | Current and past | 6 (2.6%) | 3 (2.1%) | 3 (4.2%) | 0 (0.0%) |
| ED: n(%) |  |  |  |  |  |
| AN | Current | 0 (0.0%) | 0 (0.0%) | 0 (0.0%) | 0 (0.0%) |
|  | Past | 10 (4.4%) | 8 (5.2%) | 2 (2.7%) | 5 (4.9%) |
|  | Current and past | 3 (1.3%) | 2 (1.3%) | 1 (1.4%) | 1 (1.0%) |
| BN | Current | 2 (0.9%) | 1 (0.7%) | 1 (1.4%) | 0 (0.0%) |
|  | Past | 3 (1.3%) | 2 (1.3%) | 1 (1.4%) | 1 (1.0%) |
|  | Current and past | 4 (1.8%) | 3 (2.0%) | 1 (1.4%) | 0 (0.0%) |
| BED | Current | 2 (0.9%) | 1 (0.7%) | 1 (1.4%) | 1 (1.0%) |
|  | Past | 10 (4.4%) | 7 (4.6%) | 3 (4.1%) | 2 (2.0%) |
|  | Current and past | 4 (1.8%) | 2 (1.3%) | 2 (2.7%) | 0 (0.0%) |
| Other ED | Current | 1 (0.4%) | 1 (0.7%) | 0 (0.0%) | 0 (0.0%) |
|  | Past | 7 (3.1%) | 5 (3.3%) | 2 (2.7%) | 0 (0.0%) |
|  | Current and past | 4 (1.8%) | 2 (1.3%) | 2 (2.7%) | 1 (1.0%) |
|  |  |  |  |  |  |
| Trying to lose weight: n(%) | Yes | 122 (53.7%) | 76 (49.7%) | 46 (62.2%) | 24 (47.1%) |
|  | No | 105 (46.3%) | 77 (50.3%) | 28 (37.8%) | 27 (52.9%) |
