# Supplementary material for: The short inventory of grazing (SIG): development and validation of a new brief measure of a common eating behaviour with a compulsive dimension
Source: J Eat Disord. 2019 Feb 7;7:4. doi: 10.1186/s40337-019-0234-6 (PMC6366119; doi:10.1186/s40337-019-0234-6)
Supplement: Supplementary file 3 — Additional method section: derivation of ED diagnostic groups based on the EDE-Q. (DOCX 17 kb) [file 40337_2019_234_MOESM3_ESM.docx]

**Additional File 2 – Derivation of ED diagnostic groups based on the EDE-Q**

| DSM-5 disorder and criteria | Operationalisation |
| --- | --- |
| ***EDE-Q AN n = 3*** |  |
| A Significantly low body weight  B Intense fear of weight gain/becoming fat, or persistent behaviour interfering with weight gain  C Shape/weight overvaluation | A BMI <18.5  B edeq10 (fear of weight gain) ≥ 4 OR restraint subscale score ≥ 4 OR edeq16/17/18 (vomit/laxatives/exercise) > 0  C shape concern subscale ≥ 4 OR weight concern subscale ≥ 4 |
|  |  |
| ***EDE-Q BN n = 16*** |  |
| A+C Presence of OBEs ≥1/wk for 3 mths  B+C Presence of compensatory behaviours ≥1/wk for 3 mths  D Shape/weight overvaluation  E Not AN | A+C edeq14 (overeating with a sense of LOC) ≥ 4  B+C edeq16/17/18 (vomit/laxatives/exercise) ≥ 4  D shape concern subscale ≥ 4 OR weight concern subscale ≥ 4  E Exclude cases classified as AN |
|  |  |
| ***EDE-Q BED-Broad n = 20*** |  |
| A+D Presence of OBEs ≥1/wk for 3 mths | A+D edeq14 (overeating with a sense of LOC) ≥ 4 |
| B ≥3 features associated with OBE | B N/A |
| C Marked distress regarding OBE | C N/A |
| E No compensatory behaviours | E edeq16/17/18 (vomit/laxatives/exercise) = 0 |
| F Not AN, BN | F Exclude cases classified as AN, BN |
|  |  |
| ***EDE-Q OSFED-AN n = 21*** |  |
| A Not underweight. | A BMI ≥ 18.5 |
| B Intense fear of weight gain/becoming fat, or persistent behaviour interfering with weight gain | B edeq10 (fear of weight gain) ≥ 4 OR restraint subscale score ≥ 4 OR edeq16/17/18 (vomit/laxatives/exercise) > 0 |
| C Shape/weight overvaluation | C shape concern subscale ≥ 4 OR weight concern subscale ≥ 4 |
| Not other EDs | Exclude cases classified as other EDs |
|  |  |
| ***EDE-Q OSFED-BN n = 0*** |  |
| A+C Presence of OBEs <1/wk and/or for <3 mths | A+C edeq14 (overeating with a sense of LOC) > 0 |
| B+C Presence of compensatory behaviours <1/wk and/or for <3 mths | B+C edeq16/17/18 (vomit/laxatives/exercise) > 0 |
| D Shape/weight overvaluation | D shape concern subscale ≥ 4 OR weight concern subscale ≥ 4 |
| Not other EDs | Exclude cases classified as other EDs |
|  |  |
| ***EDE-Q OSFED-BED n = 21*** |  |
| A+D Presence of OBEs <1/wk and/or for <3 mths | A+D edeq14 (overeating with a sense of LOC) > 0 |
| B ≥3 features associated with OBE | B N/A |
| C Marked distress regarding OBE | C N/A |
| E No compensatory behaviours | E edeq16/17/18 (vomit/laxatives/exercise) = 0 |
| Not other EDs | Exclude cases classified as other EDs |
|  |  |
| ***EDE-Q OSFED-PD n = 2*** |  |
| A Purging behaviour for shape- or weight-related reasons | A edeq16/17 (vomit/laxatives) > 0 |
| B No binge eating present. | B edeq14 (overeating with a sense of LOC) = 0 |
| Not other EDs | Exclude cases classified as other EDs |
