# Supplementary material for: The short inventory of grazing (SIG): development and validation of a new brief measure of a common eating behaviour with a compulsive dimension
Source: J Eat Disord. 2019 Feb 7;7:4. doi: 10.1186/s40337-019-0234-6 (PMC6366119; doi:10.1186/s40337-019-0234-6)
Supplement: Supplementary file 4 — Additional results: scores obtained by the total sample on study measures of distress, HRQoL, eating psychopathology and related constructs. (DOCX 15 kb) [file 40337_2019_234_MOESM4_ESM.docx]

**Additional File 3 - Scores obtained by the total sample on study measures of distress, HRQoL, eating psychopathology and related constructs.**

| Measure | M(SD) | Min-Max |
| --- | --- | --- |
|  | *n = 227* |  |
| Distress due to grazing | 0.60 (0.66) | 0-2 |
| DASS-21 D | 5.14 (5.26) | 0-21 |
| DASS-21 A | 3.49 (3.66) | 0-19 |
| DASS-21 S | 6.16 (4.74) | 0-20 |
| DASS-21 T | 14.79 (12.07) | 0-57 |
|  | *n = 219* |  |
| EDE-Q OOE | 4.09 (5.65) | 0-28 |
| EDE-Q OBE | 2.71 (4.54) | 0-25 |
| EDE-Q SBE | 3.35 (5.74) | 0-28 |
| EDE-Q Vomiting | 0.54 (3.40) | 0-30 |
| EDE-Q Laxative use | 0.29 (1.96) | 0-25 |
| EDE-Q Driven exercise | 3.36 (6.52) | 0-28 |
|  | *n = 226* |  |
| EDE-Q Restraint | 1.55 (1.55) | 0-6 |
| EDE-Q Eating Concern | 1.20 (1.36) | 0-5.40 |
| EDE-Q Shape Concern | 2.42 (1.72) | 0-6 |
| EDE-Q Weight Concern | 1.93 (1.59) | 0-5.80 |
| EDE-Q Global | 1.77 (1.40) | 0-5.60 |
|  | *n = 213* |  |
| BES | 11.89 (9.83) | 0-45 |
|  | *n = 210* |  |
| DEBQ Emotional | 30.06 (12.84) | 13-64 |
| DEBQ External | 30.06 (8.13) | 14-50 |
|  | *n = 215* |  |
| LOCES | 2.08 (1.08) | 1-5 |
| SHRI Total | 38.61 (19.63) | 12-84 |
| SHRI BAI | 13.02 (7.32) | 4-28 |
| GQ Grazing | 6.50 (3.95) | 0-16 |
| GQ LOC Grazing | 4.04 (3.26) | 0-12 |
| GQ Total | 10.54 (6.50) | 0-28 |
| SF-12 PCS | 51.80 (8.42) | 27.01-71.23 |
| SF-12 MCS | 40.47 (12.09) | 9.71-68.32 |
|  | *n = 209* |  |
| SDS17 (16-item) | 9.68 (3.05) | 1-16 |
