# Supplementary material for: The short inventory of grazing (SIG): development and validation of a new brief measure of a common eating behaviour with a compulsive dimension
Source: J Eat Disord. 2019 Feb 7;7:4. doi: 10.1186/s40337-019-0234-6 (PMC6366119; doi:10.1186/s40337-019-0234-6)
Supplement: Supplementary file 5 — Additional results: Differences in psychological, eating psychopathology and HRQoL scores between grazing participants with and without LOC grazing. (DOCX 18 kb) [file 40337_2019_234_MOESM5_ESM.docx]

**Additional File 4 - Differences in psychological, eating psychopathology and HRQoL scores between grazing participants with and without LOC grazing.**

|  | GRLOC-  M(SD) | GRLOC+ M(SD) | F/Welch | p | Omega-sq. |
| --- | --- | --- | --- | --- | --- |
|  | *n = 73* | *n = 137* |  |  |  |
| Age | 23.40 (8.82) | 25.61 (10.05) | 2.51 | .115 | 0.01 |
| BMI | 21.82 (3.65) | 24.13 (5.03) | 11.91 | .001 | 0.06 |
| Distress due to grazing | 0.30 (0.49) | 0.84 (0.67) | 36.84 | <.001 | 0.17 |
| DASS-21 D | 3.84 (4.67) | 5.89 (5.47) | 7.41 | .007 | 0.03 |
| DASS-21 A | 2.88 (3.49) | 3.91 (3.77) | 3.78 | .053 | 0.01 |
| DASS-21 S | 4.70 (4.02) | 6.99 (4.81) | 13.43 | <.001 | 0.06 |
| DASS-21 T | 11.41 (10.98) | 16.79 (12.37) | 9.71 | .002 | 0.04 |
|  | *n = 70* | *n = 132* |  |  |  |
| EDE-Q OOE | 1.87 (3.95) | 5.36 (6.09) | 24.22 | <.001 | 0.10 |
| EDE-Q OBE | 0.53 (1.56) | 4.17 (5.25) | 54.34 | <.001 | 0.21 |
| EDE-Q SBE | 1.23 (3.32) | 4.79 (6.51) | 26.51 | <.001 | 0.11 |
| EDE-Q Vomiting | 0.31 (2.63) | 0.73 (3.93) | 0.62 | .430 | 0.00 |
| EDE-Q Laxative use | 0.03 (0.24) | 0.47 (2.50) | 4.04 | .046 | 0.01 |
| EDE-Q Driven exercise | 2.56 (6.19) | 4.13 (6.95) | 2.52 | .114 | 0.01 |
|  | *n = 72* | *n = 137* |  |  |  |
| EDE-Q Restraint | 0.91 (1.23) | 1.96 (1.58) | 27.88 | <.001 | 0.11 |
| EDE-Q Eating Concern | 0.44 (0.56) | 1.68 (1.46) | 77.86 | <.001 | 0.27 |
| EDE-Q Shape Concern | 1.59 (1.40) | 2.98 (1.68) | 40.59 | <.001 | 0.16 |
| EDE-Q Weight Concern | 1.15 (1.22) | 2.45 (1.61) | 42.47 | <.001 | 0.17 |
| EDE-Q Global | 1.02 (0.99) | 2.27 (1.40) | 55.33 | <.001 | 0.21 |
|  | *n = 69* | *n = 128* |  |  |  |
| BES | 5.25 (4.58) | 16.3 (9.87) | 114.58 | <.001 | 0.37 |
|  | *n = 69* | *n = 125* |  |  |  |
| DEBQ Emotional | 22.54 (9.37) | 36.40 (11.89) | 79.95 | <.001 | 0.29 |
| DEBQ External | 26.20 (6.51) | 33.03 (7.65) | 39.27 | <.001 | 0.18 |
|  | *n = 70* | *n = 129* |  |  |  |
| LOCES | 1.33 (0.55) | 2.57 (1.07) | 116.55 | <.001 | 0.37 |
| SHRI Total | 30.34 (17.62) | 45.71 (17.94) | 33.71 | <.001 | 0.14 |
| SHRI BAI | 9.86 (6.35) | 15.68 (6.84) | 34.59 | <.001 | 0.15 |
| GQ Grazing | 5.04 (3.82) | 7.91 (3.40) | 29.48 | <.001 | 0.12 |
| GQ LOC Grazing | 1.67 (1.59) | 5.69 (3.07) | 148.42 | <.001 | 0.43 |
| GQ Total | 6.71 (4.61) | 13.6 (5.78) | 84.32 | <.001 | 0.30 |
| SF-12 PCS | 52.08 (8.16) | 51.86 (8.71) | 0.03 | .864 | 0.00 |
| SF-12 MCS | 44.42 (11.28) | 38.09 (12.25) | 12.78 | <.001 | 0.06 |
|  | *n = 69* | *n = 124* |  |  |  |
| SDS17 (16-item) | 10.57 (3.05) | 9.15 (2.99) | 9.86 | .002 | 0.04 |
